# A Dynamic Contour Evolution Algorithm for Cell Segmentation and Synaptic Tracking under Occlusion

**DOI:** 10.1021/cbmi.5c00227

**Published:** 2025-12-23

**Authors:** Chen-Xi Zhang, Xin-Gui Yu, Ming-Kang Li, Cheng Yang, Feng Yan, Yun Chen, Yong-Jing Wan, Yi-Tao Long, Yi-Lun Ying

**Affiliations:** † School of Pharmacy, Nanjing Medical University, Nanjing 211166, P. R. China; ‡ School of Information Science and Engineering, East China University of Science and Technology, Shanghai 200237, P. R. China; § School of Chemistry and Chemical Engineering, Molecular Sensing and Imaging Center (MSIC), Nanjing University, Nanjing 210023, P. R. China; ∥ School of Electronic Sciences and Engineering, Nanjing University, Nanjing 210023, P. R. China; ⊥ Chemistry and Biomedicine Innovation Center, Nanjing University, Nanjing 210023, P. R. China

**Keywords:** Cell segmentation, Cell tracking, FT-KAN, Dynamic contour evolution, Cell morphology, Cell migration

## Abstract

Cell migration is essential for development, tissue homeostasis, and immune regulation, and its dysregulation contributes to disease. Quantifying single-cell behaviors is a challenge due to morphological variability and occlusion. We present a robust cell tracking algorithm combining a contour deformation network, Dynamic Profile Evolution (DPE), and a graph neural network (GNN)-based framework. This method accurately segments and tracks cells under low-confidence conditions. Compared with existing approaches, it achieves improved recognition on HT22 cell data sets while maintaining high identity continuity. Analyses of over 4,000 cells reveal stable morphology and migration pattern, whereas oxidative stress reduces motility and alters trajectories. This framework enables precise quantification of cellular dynamics, providing a versatile tool for long-term live-cell monitoring and high-content analysis of cell behaviors under diverse experimental conditions.

## Graphical abstract

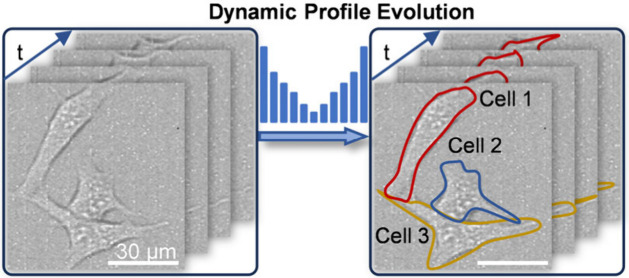

## Introduction

Cell migration, accompanied by dynamic morphological changes, is a fundamental biological process that is essential for the development and maintenance of multicellular organisms. It plays a pivotal role in tissue homeostasis, immune responses, and neurological development,^1–3^ while its dysregulation is implicated in various pathological conditions, including intellectual disability, inflammatory diseases, and cancer metastasis.^2,4–6^ A notable example is neurogenesis, where neurons must migrate from their origin to specific destinations and exhibit a rich diversity of morphologies, guided by precise temporospatial cues.^7–9^ Disruptions in this process can lead to severe neurodevelopmental disorders.^10–12^ Axon targeting further establishes the preliminary neural circuitry, which is refined through neuronal activity to form an accurate neural map.^8,13^ Understanding the mechanisms governing cell migration under both physiological and pathological conditions necessitates precise quantification of cellular behavior.^14^ Key aspects of migration analysis include tracking trajectories and assessing cellular morphology, which encompasses parameters such as cell shape, polarization, adhesion, and protrusive activity.^15–17^ However, tracking individual cells in large and dynamic populations remains a challenge due to morphological variability and transient interactions, especially in collective migration across wide imaging fields.^18^ Therefore, accurately delineating individual cell boundaries in large, dynamic populations is pivotal for extracting reliable quantitative measurements for understanding the underlying biological processes.

Neuronal morphology is considered sparse because the soma occupies only a small portion of the total volume explored by its dendritic and axonal arbors.^19^ Therefore, precise and comprehensive identification of the full spatial extent of each neuron is required. Traditional cell segmentation and tracking frameworks demonstrated notable limitations in performing consistently when cells are partially occluded.^20,21^ Uncertainty in confidence estimation can lead to occasional tracking interruptions, resulting in fragmented trajectories that may influence downstream analyses.^22^ To achieve high-precision analysis of dynamic cellular behaviors even under occlusion, we present a dynamic contour evolution approach that combines two key strategies (Figure 1). First, a contour deformation network with boundary chain code learning is coupled with a Dynamic Profile Evolution (DPE) module, enabling precise segmentation of cellular synapses and robust identification of motile cells across complex morphologies. Second, a graph neural network (GNN)-based tracking framework resolves low-confidence segmentation under occlusion.^23^ Unlike previous methods that produce few long-term continuous cell trajectories, this algorithm preserves cell identities under low confidence by updating intercellular relationships and using learnable feature correlations. Our presented approach not only ensures reliable tracking across large and dynamic populations, but also enables precise reconstruction of migration trajectories, thereby distinguishing true biological motion from imaging artifacts.

**Figure 1:**
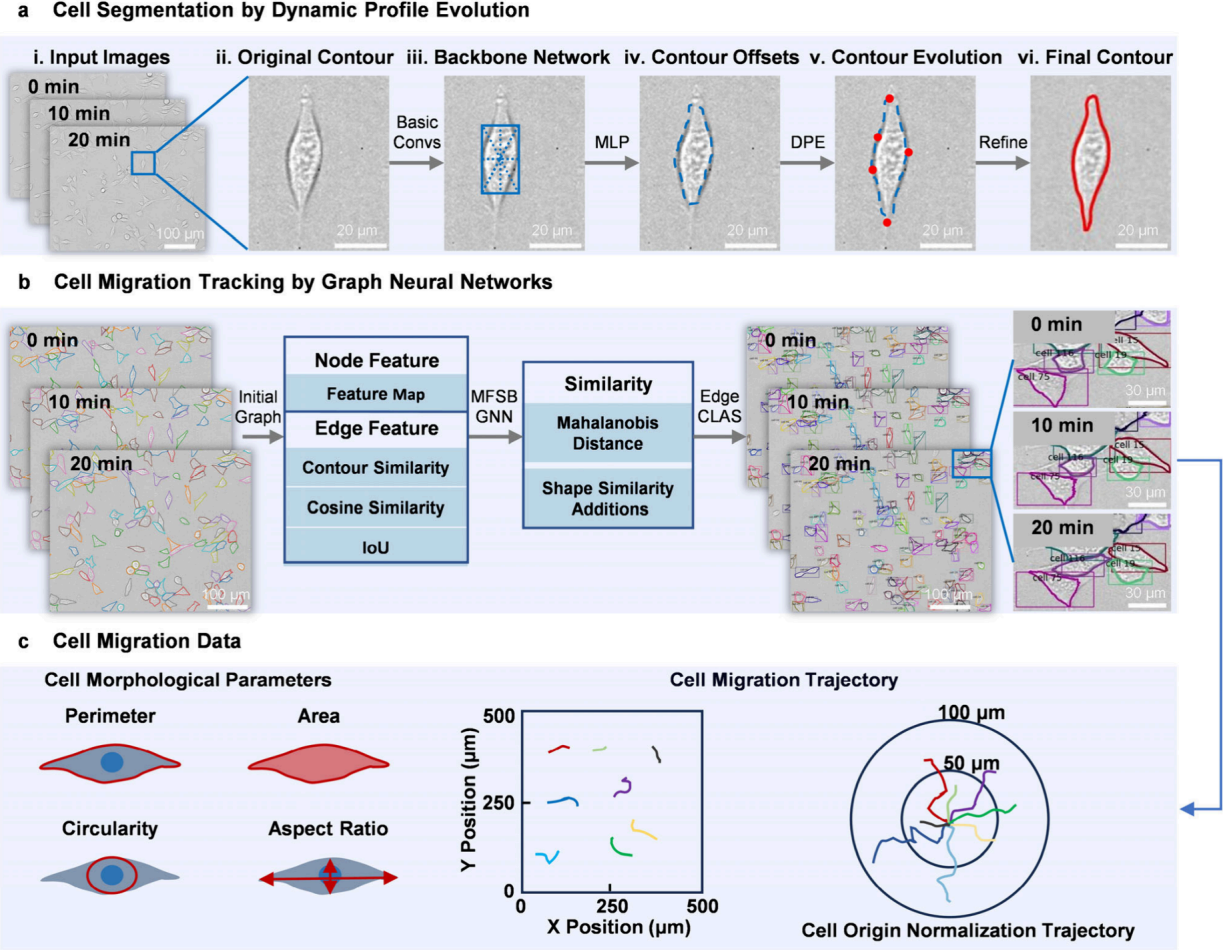
Dynamic contour evolution framework for cell segmentation and tracking. (a) Cell segmentation by Dynamic Profile Evolution. The image first undergoes basic convolutional layers (Basic Convs) for preliminary feature extraction to obtain an initial contour. These contour points are then fed into a multilayer perceptron (MLP) model, which computes offset vectors for each point to iteratively refine the contour position. For precise identification of synaptic structures within cells, the Dynamic Profile Evolution (DPE) algorithm is applied to extract key cellular landmarks. The vertices of the initial contour are adaptively stretched or contracted toward these landmarks, followed by a final contour refinement step to produce the optimized boundary. (b) Cell tracking and target matching. (1) Initial Graph: High-confidence cell targets from consecutive frames are represented as nodes, with edges encoding the appearance, contour, and spatial similarity. (2) MFSB-GNN: A Multifeature Similarity Boosted Graph Neural Network updates node and edge features by propagating contextual information to enhance matching confidence. (3) Edge CLAS: An Edge Confidence Learning and Aggregation Strategy computes the final matching probability for each edge, enabling robust identity preservation under occlusion. (c) Quantitative analysis of cell migration. The integrated cell segmentation and tracking algorithm enables the extraction of morphological parameters, including perimeter, area, circularity, and aspect ratio, and trajectories for individual cells.

## Experimental Section

### Live-Cell Imaging

HT22 cells, a mouse hippocampal neuronal cell line, were purchased from Wuhan Saios Biotechnology Co., Ltd. BV2 cells, a mouse microglial cell line, were obtained from the Cell Bank of Chinese Academy of Sciences. HT22 and BV2 cells were cultured in Dulbecco’s Modified Eagle Medium supplemented with 10% fetal bovine serum and 1% penicillin-streptomycin at 37 °C and 5% CO_2_. HT22 hippocampal neuronal cells were treated with 10 μM sodium arsenite for 24 h after adherence to induce oxidative stress. Cell suspensions were collected and transferred to a lensless shadow microscopic imaging system (Nanjing Gotrium Science and technology Co., Ltd.) for live-cell imaging. Images of cells were captured at 10 min intervals.

### Model Training and Implementation

The model was implemented using the PyTorch framework and trained and evaluated on a NVIDIA GeForce RTX 4090 GPU (produced by NVIDIA Corporation). Training was performed with a batch size of 4 over 200 epochs. The Adam optimizer was employed, with an initial learning rate *L*_0_ = 1 × 10^–5^. A learning rate decay strategy was applied every 50 epochs according to the equations as follows

$$L_{i+1}{=L}_{i}\times \gamma$$

where *L*_*i*_ is the learning rate at the epoch *i*, and γ is 0.1. To prevent overfitting, a random dropout strategy was applied during the training.

### Cell Segmentation and Tracking Algorithm

#### Cell Images

Both control and experimental group cells were cultured under standard conditions (see details in the Cell Culture and Experiment section in the SI) and continuously imaged over a 20-h period. Images were acquired at 10 min intervals, yielding a total of 121 images per group.

#### Segmentation

The detection and segmentation process aims to accurately segment individual cells in each frame, followed by verification during the tracking phase. Figure 1 illustrates the overall framework for cell instance segmentation. The backbone network of the algorithm incorporates adaptive convolution kernels based on Kolmogorov-Arnold Network (KAN) and Fourier Transform Kolmogorov-Arnold Network (FT-KAN) modules. This enables the dynamic adjustment of image receptive fields, allowing for better adaptation to cellular features of varying scales and shapes, thereby enhancing the extraction of fine-grained features.

To further enhance the accuracy of segmentation contour boundaries, a Boundary Feature Enhancement (BFE) module is connected after the backbone network. This module is specifically designed to generate the initial contour, ensuring refined edge details and improved segmentation precision. The module performs learnable contour offset based on boundary chain codes and features. By encoding cellular edge contour information and dynamically adjusting boundary positions, it not only enriches contour feature representations but also ensures the coherence of cellular boundaries, achieving both enhanced detail capture and structural integrity. To address the challenges of segmenting cellular synaptic boundaries and achieve optimal segmentation results, a DPE module is designed to refine the initial contours. The DPE module employs an iterative optimization strategy to progressively adjust the predicted cell contours until convergence. Finally, contour postprocessing steps, including smoothing and noise removal, are applied to obtain the final segmentation results.

#### Tracking

The cell tracking algorithm is developed based on the Multi-Feature Spatial Similarity-Boosted Graph Neural Network, which aims to achieve multiobject tracking of cell instances through the construction and iterative updating of a graph network (Figure S1). The cell tracking process begins by scoring the segmented cell images and selecting the top K (Top-K) segmentation results with the highest scores for further processing. To achieve matching between cell targets, node features and edge features are defined. Node features typically include appearance features extracted by deep learning models, while edge features involve more specific relational information between cell targets, such as contour similarity, cosine similarity of feature vectors, and the degree of overlap between targets (i.e., Intersection over Union of cells, IoU^24^). The initial equation for edge features is as follows:

$$e_{i,j}^{0}=f\left({\mathrm{IoU}}_{i,j},{\mathrm{Sim}}_{i,j},{\mathrm{Contour}}_{i,j}\right)$$

Here, Contour_*i*,*j*_ represents the contour similarity between two cells, IoU_*i*,*j*_ represents the IoU value between cells, and Sim_*i*,*j*_ represents the cosine similarity between cells. *e*_*i*,*j*_^0^ and *e*_*j*,*i*_^0^ are distinct features, and *f*(·) denotes a fully connected layer.

These features collectively form the basis for measuring the similarity between the two cell targets. After initialization, the network updates this bipartite graph by modifying both nodes and edges. This process employs a similarity-enhancement strategy to increase the connection weights between potential matching targets. Specifically, edge weights are adjusted by calculating the sum of the Mahalanobis distance and cell shape similarity, thereby increasing the matching probability for targets likely representing the same cell across consecutive frames. The equation is listed as follows

$$e_{i,j}^{t}=f_{e}\left(e_{i,j}^{0},e_{i,j}^{t-1},v_{i}^{t-1},v_{j}^{t},{\mathrm{MhD}}_{i,j},{\mathrm{shape}}_{i,j}\right)$$

where MhD_*i*,*j*_ represents the Mahalanobis distance between nodes and is calculated as follows

$${\mathrm{MhD}}_{i,j}={\left({\left(v_{i}^{t-1}-v_{j}^{t}\right)}^{T}\sum \left(v_{i}^{t-1}-v_{j}^{t}\right)\right)}^{1/2}$$

where shape_*i*,*j*_ represents the sum of cell shape similarities, *v*_*i*_^*t*–1^ and *v*_*j*_^*t*^ are feature vectors of cells from consecutive frames, and *f*_e_(·) denotes a fully connected layer.

Finally, the updated edge similarity score *S*_sim_ is compared with a predefined threshold *S*_τ_. If *S*_sim_ > *S*_τ_, the corresponding cell targets in the two frames are considered successfully matched.

## Results and Discussion

To enable the accurate quantification of single-cell behavior under conditions of morphological variability and occlusion, we first developed and evaluated the proposed DPE module for cell segmentation. The results compared with five previous reported algorithms, including pixel-based architectures of Mask Region-based Convolutional Neural Network (Mask RCNN),^25^ contour-optimized architectures of DeepSnake^26^ and End-to-End Contour-based Instance Segmentation (E2EC),^27^ single-shot detection of YOLOv8,^28^ and Transformer-based architectures of Polygon Transformer (Poly-Former),^29^ demonstrates substantial performance gains on HT22 cell data sets. Specifically, our approach achieves a 66.5% absolute increase in Average Precision (AP)^30^ at an IoU threshold of 0.5 (AP_50_) and a 49.3% improvement in Average Recall (AR)^31^ at mid-range IoU thresholds (AR_mid_) (Table S1). Here, AP_50_ quantifies detection accuracy, while AR_mid_ reflects the ability to recover target cell instances under moderate overlap conditions. Such improvements result from the model’s hierarchical feature integration, enabling precise preservation of cellular morphology during multiscale processing. This framework also captures spatial relationships between cells, addressing a key limitation of conventional methods, where low-confidence detections yield incomplete features. Empirical validation confirms the framework’s robustness in cases of partial occlusion or ambiguous boundaries, as it consistently tracks masked cell instances across sequential frames (Figure 2a). These advancements enhance the precision of automated cell behavior analysis, particularly in biomedical applications requiring long-term trajectory integrity for motility quantification.

**Figure 2:**
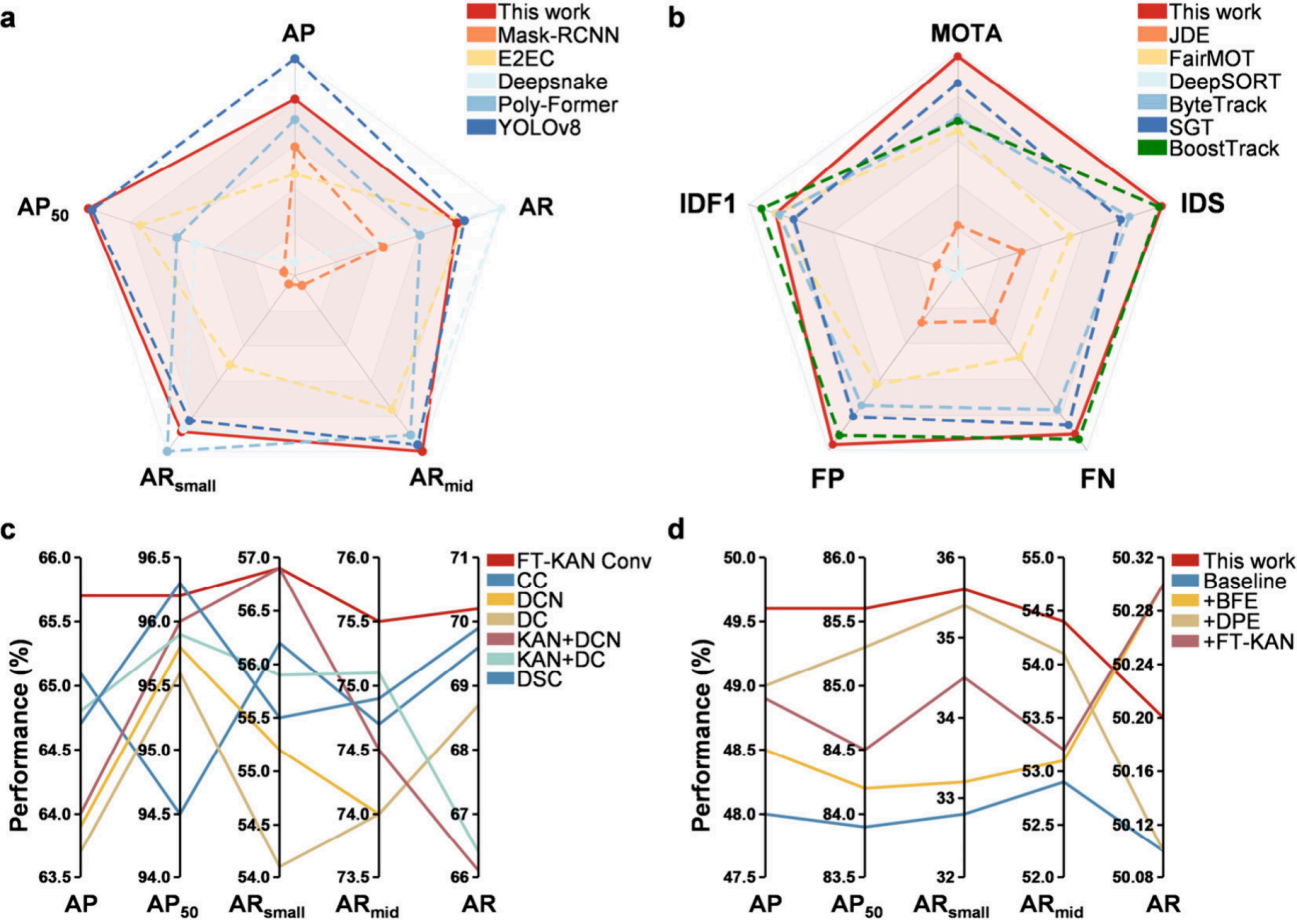
Comprehensive evaluation of the proposed Dynamic Contour Evolution framework for cell segmentation. (a) Comparison of cell segmentation models. Performance comparison of different cell segmentation models on HT22 cell data sets including this work, Mask R-CNN (Mask Region-based Convolutional Neural Network), E2EC (End-to-End Contour-based Instance Segmentation), Deepsnake, Poly-Former (Polygon Transformer) and YOLOv8 (You Only Look Once: Version 8) based on AP (Average Precision), AP_50_ (Average Precision at IoU = 0.5), AR (Average Recall), AR_mid_ (Average Recall at mid-range IoU thresholds), and AR_small_ (Average Recall for small objects). (b) Evaluation of cell tracking algorithms. Comparative performance of this work with JDE (Joint Detection and Embedding), FairMOT (Fair Multi-Object Tracking), DeepSORT (Deep Simple Online and Realtime Tracking), ByteTrack, SGT (Spatial-Temporal Graph Tracking) and BoostTrack, bsed on MOTA (Multiple Object Tracking Accuracy), IDS (Identity Switches), FN (False Negative), FP (False Positive), and IDF1 (ID F1 Score) metrics. (c) Effect of convolutional kernel designs. Comparison among different convolution types including FT-KAN Conv (Fourier Transform Kolmogorov-Arnold Network Convolution), CC (Classical Convolution), DCN (Deformable Convolutional Network), DC (Dilated Convolution), KAN+DCN, KAN+DC and DSC (Dynamic Snake Convolution). (d) Model optimization and ablation analysis. The experiments were conducted using the E2EC algorithm as the baseline to evaluate the model’s performance under different module configurations. When the DPE (Dynamic Profile Evolution), BFE (Boundary Feature Enhancement), and FT-KAN modules were all integrated into the framework (this work), the model achieved optimal performance.

In dense cell scenarios, cell adhesion and occlusion frequently occur, significantly increasing the difficulty of cell detection, segmentation, and tracking. An ideal cell tracking system should possess the capability to efficiently and accurately track all targets in a video while maintaining a consistent identity continuity for each target. To evaluate the tracking performance, comparative experiments were conducted using different models on HT22 cell data sets (Figure 2b). HT22 cells typically exhibit a spindle-shaped morphology characterized by stable shapes and well-defined boundaries. The results show that our proposed model outperforms existing methods in key tracking metrics, achieving a Multiple Object Tracking Accuracy score (MOTA)^32^ of 75.7%, with 410 identity switches (IDS)^33^ and 310 false positives (FP)^34^ (Table S2). The lowest IDS value is particularly crucial as it ensures identity continuity throughout the observation period, providing a solid foundation for precise analysis of subsequent cell migration morphological indicators (e.g., migration paths, velocity distributions). These data indicate that the model can accurately and efficiently identify and continuously track a large number of moving cells.

BV2 cells exhibit an elongated or irregular morphology characterized by dynamic remodeling of protrusions. Given the elongated shape and rapid morphological dynamics of BV2 cells, the segmentation process requires a higher precision in boundary delineation. Therefore, we examined the impact of different convolutional architectures on the accuracy and robustness of synaptic segmentation. On the BV2 cell data set, the impact of different convolution techniques on cell synapse segmentation was assessed. Experimental results reveal that variations in convolutional design significantly influence performance metrics (Table S3). Among the tested architectures, KAN Convolution exhibited superior performance, particularly in AP and AR for small objects (AR_small_). Figure 2c illustrates the segmentation results of BV2 cell synapses using various convolution methods, where the FT-KAN Conv-based model demonstrates the highest precision and finest synapse segmentation quality.

To further refine the model, we conducted ablation experiments to assess the contribution of key components, including the BFE module, the DPE module, and the FT-KAN. Experimental results indicate that the integration of the three modules yields superior overall performance, while providing notable advantages in addressing targets across multiple scales (Table S4). This joint framework leverages the complementary strengths of each module, leading to consistent improvements in accuracy and reliability under diverse evaluation settings (Figure 2d).

Based on the developed cell segmentation and tracking algorithms, we established a standardized quantitative evaluation framework to systematically characterize the images from HT22 hippocampal neuronal cells. We quantitatively assess 4,141 cells across four morphological parameters of perimeter, area, circularity, and aspect ratio. The perimeter distribution exhibited a pronounced peak around 100 μm, with values predominantly ranging from 80 to 100 μm, indicating a relatively homogeneous cell population in overall cellular dimensions (Figure 3a). Similarly, the area parameter exhibited a Gaussian-like distribution centered near 300 μm^2^, ranging from 100 to 700 μm^2^ (Figure 3b). This result suggests consistent cellular spreading behavior under the experimental conditions. Circularity measurements revealed a distribution with a modal value near 0.5, spanning from 0.3 to 0.7 (Figure 3c). These results indicate that a majority of cells adopted moderately elongated morphologies rather than perfect circularity. The aspect ratio distribution peaked between 0.4 and 0.5 within a 0.2–1.0 range, further supporting the observed moderate elongation (Figure 3d). These parameters collectively suggest that HT22 cells maintain sufficient spread for functional processes while retaining morphological plasticity. The statistical consistency observed across all four parameters underscores the reproducibility of cellular adhesion and spreading on the chip substrate.

**Figure 3:**
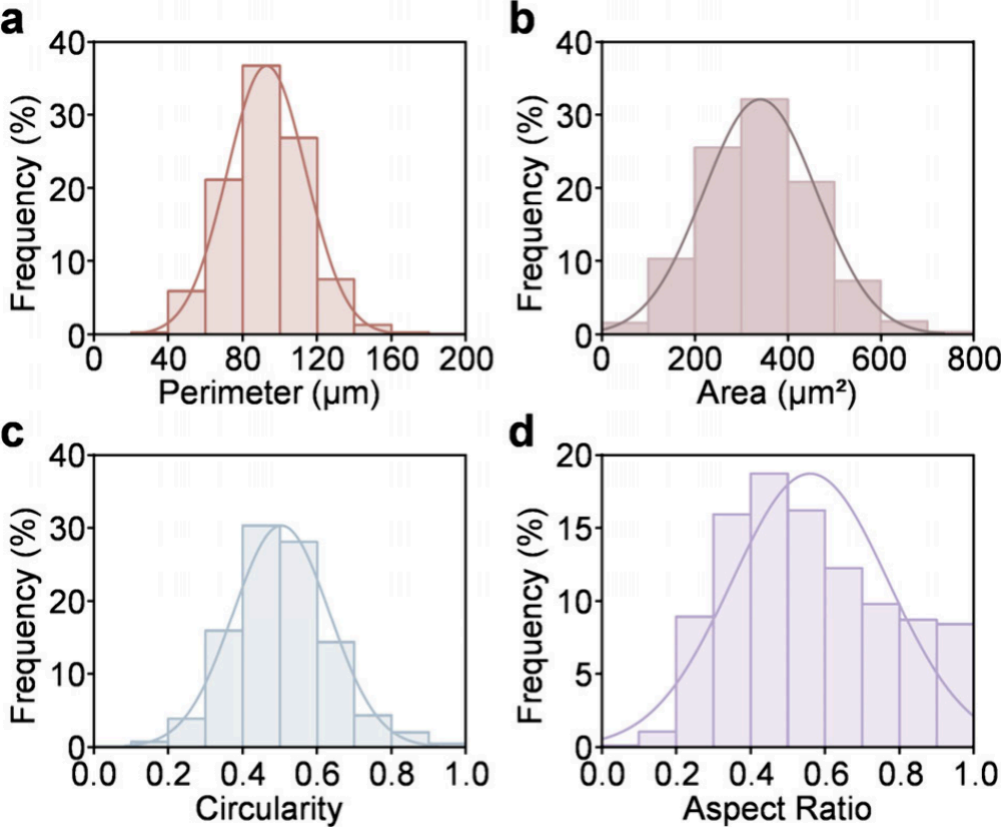
Normal distribution plots of morphological parameters for HT22 cells adherently grown on microchip surfaces, showing cell perimeter (a), area (b), circularity (c), and aspect ratio (d). The total number of analyzed cells is indicated (*N* = 4141).

To evaluate the effect of oxidative stress on the neuronal migration, we tracked HT22 cell trajectories over a 20 h period, with visualization results shown in Figure 4. 3D trajectory plots of the control group exhibited disorganized movement patterns, while the cell origin normalization trajectories demonstrated pronounced irregularities with a wider distribution range (Figure 4a). The majority of trajectory lengths clustered around 60 μm, indicating robust cell viability. In contrast, sodium-arsenite-treated cells exhibited shorter migration distances and more regular movement patterns (Figure 4b). Trajectories were more uniformly distributed and predominantly clustered near 40 μm. These results indicate that oxidative stress significantly alters neuronal cell migration trajectories, modifies spatial movement patterns, and reduces cell motility.

**Figure 4:**
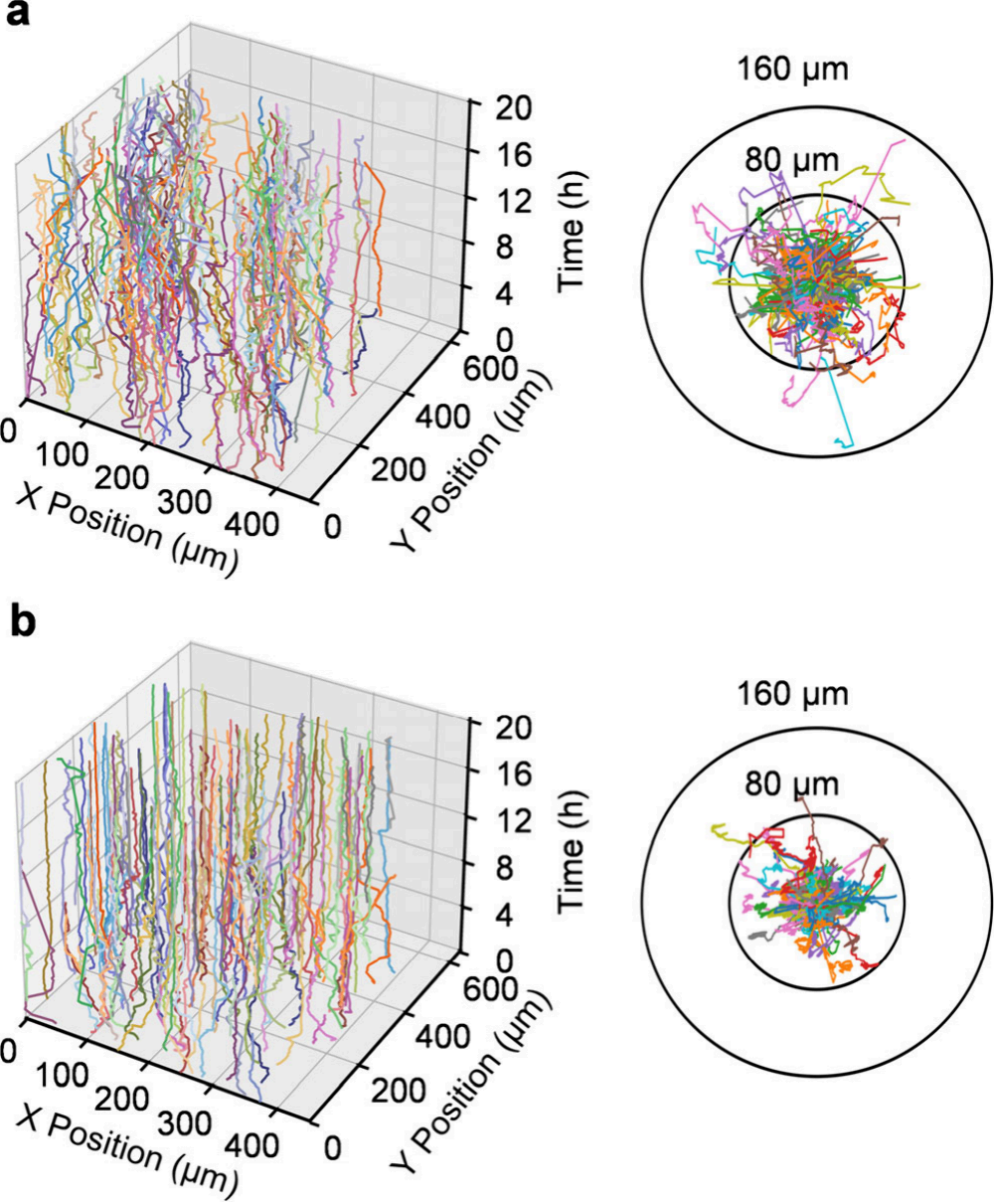
Three-dimensional cellular trajectory plots and cell origin normalization trajectories for HT22 cells under normal conditions (a, *N* = 184) and oxidative stress (b, *N* = 143).

## Conclusions

In summary, we developed a novel and robust cell tracking algorithm capable of accurately identifying and tracking cells under complex morphologies and partial occlusion. The algorithm integrates a detection network with a contour deformation module based on boundary chain code learning together with a DPE module for robust identification of motile cells. A module-based tracking strategy then links cells across sequential frames, preserving identity continuity and minimizing trajectory fragmentation, even under challenging conditions. Experimental results demonstrate consistent and enhanced performance across multiple evaluation metrics for both segmentation and tracking, confirming the method’s suitability for high-precision cell migration analysis. Therefore, our cell image analysis algorithm enables the detailed characterization of cellular responses, producing standardized morphological profiles that reveal subtle phenotypic changes during long-term live-cell monitoring. These quantitative metrics link cell morphology, migration dynamics, and functional outcomes, supporting more accurate correlations between morphological alterations and cellular functions during long-term live-cell monitoring.

## Supplementary Material



## Notes

Cell culture and experiment; cell dynamic contour evolution algorithm; graph network cell tracking algorithm with spatial similarity enhancement; validation of the cell algorithm (PDF)
