# Supplementary material for: A Dynamic Contour Evolution Algorithm for Cell Segmentation and Synaptic Tracking under Occlusion
Source: Chem Biomed Imaging. 2025 Dec 23;4(5):760–6. doi: 10.1021/cbmi.5c00227 (PMC13217357; doi:10.1021/cbmi.5c00227)
Supplement: Supplementary file 1 [file im5c00227_si_001.pdf]

Supporting Information

A Dynamic Contour Evolution Algorithm for Cell Segmentation and Synaptic Tracking under Occlusion

Chen-Xi Zhang[a]†, Xin-Gui Yu[b]†, Ming-Kang Li[c], Cheng Yang[d*], Feng Yan[d], Yun Chen[a*], Yong-Jing Wan[b*], Yi-Tao Long[c], and Yi-Lun Ying[c,e*]

a. School of Pharmacy, Nanjing Medical University, Nanjing 211166, P. R. China. *Email: ychen@njmu.edu.cn

b. School of Information Science and Engineering, East China University of Science and Technology, Shanghai 200237, P. R. China. *Email: wanyongjing@ecust.edu.cn

c. School of Chemistry and Chemical Engineering, Molecular Sensing and Imaging Center (MSIC), Nanjing University, Nanjing 210023, P. R. China. *Email: yilunying@nju.edu.cn

d. School of Electronic Sciences and Engineering, Nanjing University, Nanjing 210023, P. R. China. *Email: cyang@nju.edu.cn

e. Chemistry and Biomedicine Innovation Center, Nanjing University, Nanjing 210023, P. R. China

† These authors contributed equally to this work.

Experimental Sections

Cell culture and experiment

HT22 cells, a mouse hippocampal cell line, were cultured at 37℃ under 5% CO_2 in Dulbecco's Modified Eagle Medium supplemented with 10% fetal bovine serum and 1% penicillin-streptomycin. HT22 cells were seeded onto the chip surface at a density of 5,000 cells per well and allowed to adhere for 12 hours. Following adhesion, the experimental group was treated with 2 mL of 10 μM sodium arsenite solution, while the control group received an equal volume of PBS. Time-lapse imaging of cellular morphology was initiated immediately under standard culture conditions (37°C, 5% CO_2) with images captured at 10-minute intervals.

Cell Dynamic Contour Evolution Algorithm

Images are first processed by the Basic Convolution modules, which perform the initial feature extraction from raw cellular inputs. The resulting feature maps then undergo downsampling operations to reduce spatial dimensions while simultaneously increasing channel depth, thereby enabling deeper exploration of cellular image information. These processed feature maps are subsequently passed into residual blocks, where high-level semantic features are further extracted and enhanced.

Within this framework, the Boundary Feature Enhancement (BFE) module plays a pivotal role in constructing the initial contour evolution. This module integrates the Boundary Chain Code (BCC) representation with the extracted feature maps to achieve channel dimension reduction and feature transformation, converting coarse contour points into more precise boundary representations. Specifically, a 1×1 convolutional layer is first applied to the input feature maps to reduce channel dimensionality, thus lowering computational complexity associated with the large number of contour coordinates. The reduced features are then processed by a Multi-Layer Perceptron (MLP) to compute offset vectors for each contour point. The BCC is a coding methodology for polygonal boundaries that represents object outlines using interconnected line segments with fixed length and orientation. By encoding directional changes between successive contour points, BCC enables systematic and compact contour representation. To effectively integrate this representation into the learning process, each cellular contour in the input image is encoded using BCC, which is then incorporated as supplementary features into the BFE module.

After passing through the backbone network, the cellular image produces an initial contour, which undergoes a series of contour evolution steps. The first step is contour offsetting, in which contour points are input into an MLP to compute offset vectors using learnable weights and biases. These offsets are added to the original contour points to adjust their positions, achieving the first stage of contour evolution.

Initially, the traditional Douglas-Peucker algorithm is applied to the original image for polygon fitting. The specific steps are as follows: Take two consecutive points P_1 and P_2 from the label as the start and end points, calculate the distance from all other contour points to the line segment P_1P_2, and find the point P_{m1} with the maximum distance d_m. If d_m is less than a threshold, discard all intermediate points. Otherwise, retain P_{m1}, then repeat the previous step for the segments P_1 and P_{m1}, and P_2 and P_{m1}, until all significant points P_{mn} are identified and treated as key points in the contour.

Subsequently, during the contour fitting process, the initial contour map and the key point map are combined. Points that have not reached the contour boundary are pulled towards the key points. The operation is performed as follows: Calculate the distances between several points nearest to each contour key point and the key points themselves, and minimize the loss value to bring these points closer to the key points. The formula for calculating $Loss_{key}$ is as follows:

$$Loss_{key} = \frac{1}{N}\sum_{i=1}^{N}\sum_{j=1}^{n} \|pre_{i,j} - key_i\|$$

key_i represents the i-th key point, and $pre_{i,j}$ represents the j-th predicted contour point near the i-th key point. N denotes the number of key points reserved for the contour. Finally, each contour undergoes one last refinement step. The formula for calculating $Loss_{ref}$ is as follows:

$$Loss_{ref} = \frac{1}{N}\sum_{i=1}^{N} \left\|x_i^{pred} - x_i^{gt}\right\|$$

Here, x_i^{gt} represents the ground truth label value, and x_i^{pred} represents the predicted contour point. This final refinement step is indispensable, as the preceding key-point attraction process may displace some contour points from their true positions. By performing refinement last, the method ensures the evolved contour converges toward the optimal boundary configuration. The fused contour and BCC features are then processed by an MLP to aggregate information across contour points. Subsequent convolutional operations capture spatial dependencies and local geometric patterns by sliding kernels along the contour, thus generating higher-order feature representations. Finally, channel dimension reduction is applied to compress these high-dimensional features, enabling contour evolution based on refined representations and producing the final, precise cellular contour.

Graph Network Cell Tracking Algorithm with Spatial Similarity Enhancement

This study proposes a graph neural network-based cell tracking algorithm enhanced by spatial similarity, named MFSB-GNN (Multi-Feature Spatial Similarity-Boosted Graph Neural Network). The algorithm employs MFSB-GNN as its core framework and achieves multi-object cell instance tracking through three stages: initial graph construction, iterative graph updating, and edge classification with association matrix generation. In the initial graph construction stage, the algorithm extracts node features composed of appearance information obtained from the instance segmentation model, as well as edge features from the segmented cell instances. The edge features integrate multiple spatial and appearance cues, including the interframe cell center distance (computed from the centroid coordinates of cells in consecutive frames), cosine similarity (calculated from the feature vectors of cell nodes), and the boundary intersection-over-union (BIoU). The BIoU not only reflects the overlap between cell regions but also incorporates the boundary distance between cells across frames and the aspect ratio of their bounding boxes. Together, these features provide a comprehensive spatial similarity representation, forming a robust foundation for subsequent graph updates and association processes.

After completing the initialization, the network will further update this bipartite graph, including the update of nodes and edges. The graph network structure MFSB-GNN in this paper is composed of L FSB-MPNNs connected together, where L is the number of iterations to connect the relationships of cell instances between consecutive frames.

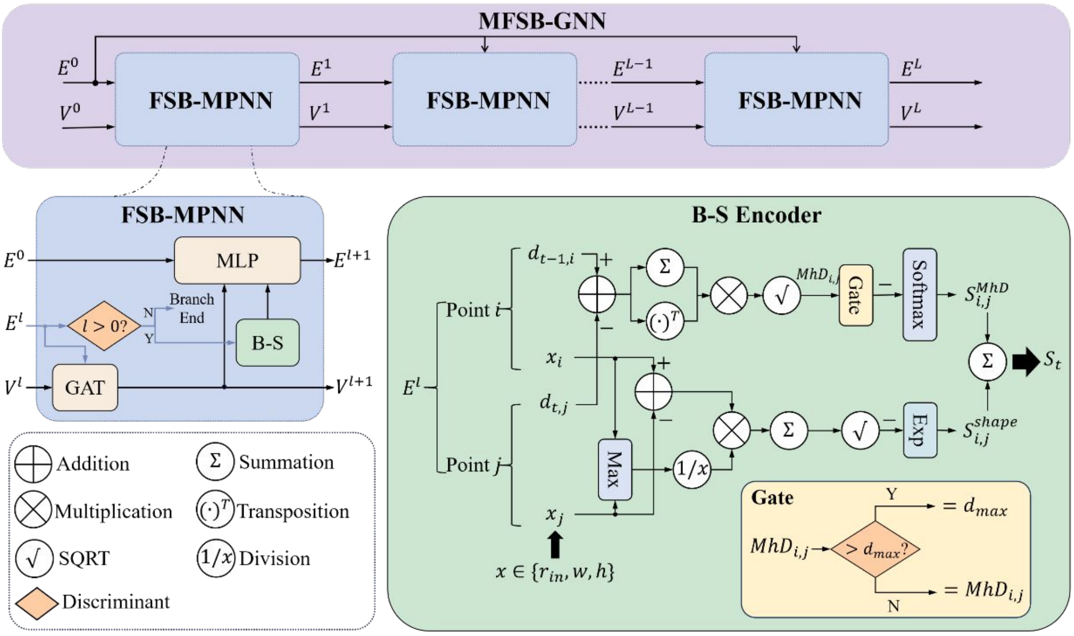

Figure S1. MFSB-GNN Network Architecture

The initial input of the network is the node feature vector V^0 and edge feature vector E^0 of cells. The node features are updated by introducing an attention mechanism, while the edge features are fed into the B-S Encoder through the updated node features V^1 and the initial edge features E^0 to obtain the updated edge features E^1. The update of node features is shown in equation:

$$v_i^1 = f_{agg,j\in\delta(i)\cup\{i\}}(f_{edge}\left(e_{i,j}^0\right)f_{node,0}\left(v_i^0\right)$$

where $f_{agg,j\in\delta(i)\cup\{i\}}$ is an aggregation function, and the summation function is selected through experiments. $\delta(i)$ denotes the nodes in the neighborhood of node i, which can be obtained from the initial graph network. $f_{edge}(\cdot)$ represents the weighted attention parameter via edge features, and $f_{node,0}(\cdot)$ denotes a fully connected layer.

For the update of edge features, a B-S Encoder is employed, as shown in Figure S1. This encoder introduces two spatial similarity measurement methods: Mahalanobis distance and shape similarity measure. When cells are occluded, they may still be recognized by the algorithm. At this time, the target is only partially visible, and its detection confidence is low, which is below a certain threshold, making it prone to missed detection or false detection. Meanwhile, during the cell division process, new cell nodes will be regarded as outliers in the network, leading to their erroneous discard and affecting the continuity and integrity of tracking. To address this issue, this paper introduces the Mahalanobis distance as a similarity measurement method, which is used to measure the difference between a point and a distribution or between two random variables from the same distribution, thereby improving the model's robustness to occlusion and division scenarios. The Mahalanobis distance formula between two points is shown in equation:

$$MhD_{i,j} = \left(\left(d_{t-1,i} - d_{t,j}\right)^T \sum \left(d_{t-1,i} - d_{t,j}\right)\right)^{\frac{1}{2}}$$

where $\sum(\cdot)$ is the covariance matrix, and $d_{t-1,i}$ and $d_{t,j}$ represent the coordinates of two points in consecutive frames, respectively. According to the formula, the Mahalanobis distance follows a chi-square distribution, which considers the correlation between features and measures based on the overall covariance structure of

the data. Therefore, it can more accurately reflect the true differences of cells in multi-dimensional features such as position and shape. However, phenomena such as cell occlusion and cell division are likely to occur during tracking, causing changes in edge features and resulting in abnormally large Mahalanobis distances. If such excessively large distance values are not restricted, the probability of false matching will increase. To handle outliers, a threshold d_{max} is set to filter out appropriate distances. The adjusted Mahalanobis distance is shown in equation:

$$MhD_{i,j} = \begin{cases} d_{max} \; if \; MhD_{i,j} > d_{max} \\ MhD_{i,j} \quad otherwise \end{cases}$$

By setting d_{max}, some abnormally matched targets can be effectively filtered out, reducing the probability of false matching. Finally, normalization is performed through the Softmax function to obtain the similarity score $S_{i,j}^{M\text{h}D}$, as shown in equation:

$$S_{i,j}^{M\text{h}D} = \text{Softmax}\left(-MhD_{i,j}\right)$$

When two cells are highly overlapping, the corresponding cell trajectory may have a higher BIoU with an incorrect detection box, thus leading to ID switch. To alleviate the ambiguity problem of similarity measurement in dense cell scenarios, this paper introduces a shape similarity measurement based on cell instance segmentation. Considering that the shape variation of the same cell in adjacent frames is usually small, its morphological indicators should remain relatively stable. Cell contour information can serve as a matching basis, helping to reduce the ambiguity in the ID assignment process and improve tracking accuracy. Meanwhile, when the confidence of detection results is low, directly applying them to shape similarity comparison may decrease the matching reliability. Therefore, a confidence weighting mechanism is introduced to further enhance the robustness of the algorithm in complex scenarios, and the confidence weighting parameter is shown in equation:

$$c_{i,j} = \begin{cases} c_{t,i} \cdot c_{t+1,j}, & if \; \text{IoU}\left(c_{t,i}\right) > \tau_{\text{I}_0\text{U}} \; and \; \text{IoU}\left(c_{t,i}\right) > \tau_{\text{I}_0\text{U}} \\ 0, & otherwise \end{cases}$$

where $c_{t,i}$ and $c_{t+1,j}$ are the confidence scores of inter-frame cells, and， $\tau_{\text{I}_0\text{U}}$ is the IoU threshold. The specific metric formulas $S_{i,j}^{shape}$ are given in equations:

$$shape_{i,j} = \sqrt{\left(\frac{r_{in,}i - r_{in,j}}{max\left(r_{in,}i,r_{in,j}\right)}\right)^2 + \left(\frac{wi - w_j}{max\left(wi,w_j\right)}\right)^2 + \left(\frac{hi - h_j}{max\left(hi,h_j\right)}\right)^2}$$

$$S_{i,j}^{shape} = c_{i,j} \exp\left(-shape_{i,j}\right)$$

Here, $shape_{i,j}$ denotes the shape difference between two nodes, $r_{in,}i$ and $r_{in,}j$ represent the distances from the centroids of the two nodes to the farthest contour point, and w and h stand for width and length, respectively. The total similarity score St is expressed as equation:

$$St = \theta_1 S^{Mh}(D_{t-1},D_t) + \theta_2 S^{shape}(D_{t-1},D_t)$$

where θ_1 and θ_2 are hyperparameters to balance the two modules. This process adopts a similarity enhancement strategy to increase the connection weight between potential matching targets. The B-S module adjusts edge weights by calculating the sum of Mahalanobis distance and cell shape similarity, so that instances of the same cell in different frames have a higher matching probability. The corresponding edge feature update is shown in equation:

$$e_{i,j}^0 = f_e\left(e_{i,j}^0, v_i^0, v_j^0, S_0\right)$$

where $f_e(\cdot)$ represents a multi-layer perceptron. For edge feature update, fusion with the initial edge feature is needed, as in equation:

$$e_{i,j}^t = f_e\left(e_{i,j}^0, e_{i,j}^{t-1}, v_i^{t-1}, v_j^t, S_t\right)$$

Finally, by comparing the updated similarity S with the preset threshold S_τ, if the association score exceeds S_τ, the corresponding cell targets in the two frames are considered successfully matched. In dense cell scenarios, cell adhesion and occlusion often occur, which increases the difficulty of cell detection and segmentation. Even with high-performance detectors, targets cannot be detected without contextual information, making it impossible to match the cell connection relationship between two frames. However, cells with the same ID have a fixed relative spatial relationship and similar motion features between adjacent frames. Therefore, this constraint is used to perform secondary detection on cells to reduce misjudgments of occluded cells.

Validation of the cell algorithm

To validate the performance of the cell tracking algorithm, we conducted comparative experiments with other existing cell tracking algorithms.

Table S1 Comparison of cell segmentation algorithms on HT22 cells

Model	AP	AP_{50}	AR_{small}	AR_{mid}	AR
ours	49.6%	85.6%	35.6%	54.4%	50.2%
Mask-RCNN[1]	38.9%	80.3%	30.3%	49.3%	49.2%
E2EC[2]	48.5%	84.2%	33.2%	53.1%	50.3%
DeepSnake[3]	47.2%	82.7%	35.5%	54.1%	50.8%
Poly-Former[4]	49.3%	83.2%	36.3%	53.9%	49.7%
Yolo-v8[5]	50.2%	85.5%	35.2%	54.2%	50.3%

Table S2 Cell tracking metrics of HT22 cells

Method	MOTA(%)	IDF1(%)	FP	FN	IDS
JDE[6]	61.9	50.3	523	2802	685
FairMOT[7]	69.6	60.3	415	2574	590
DeepSORT[8]	59.7	49.7	601	3088	801
ByteTrack[9]	70.7	60.1	379	2453	473
SGT[10]	73.5	59.2	359	2356	490
BoostTrack[11]	70.4	61.2	326	2268	415
Ours	75.7	60.3	310	2301	410

Table S3 Evaluation of different convolution kernels on BV2 cells

Method	AP	AP_{50}	AR_{small}	AR_{mid}	AR
KAN Convolution[12]	65.7%	96.2%	56.9%	75.5%	70.2%
Classical Convolution	65.1%	94.5%	56.2%	74.7%	69.6%
Deformable Convolution[13]	63.9%	95.8%	55.2%	74.0%	68.7%
Dilated Convolution[14]	63.7%	95.6%	54.1%	74.0%	68.7%
KAN+DCN	64.0%	96.0%	56.9%	74.5%	66.1%
KAN+DC	64.8%	95.9%	55.9%	75.1%	66.4%

DSC[15]	64.7%	96.3%	55.5%	74.9%	69.9%

Table S4 Evaluation of different modules on HT22 cells

Method	AP	AP_{50}	AR_{small}	AR_{mid}	AR
Ours	49.6%	85.6%	35.6%	54.4%	50.2%
Baseline	48.0%	83.9%	32.8%	52.9%	50.1%
+BFE	48.5%	84.2%	33.2%	53.1%	50.3%
+DPE	49.0%	85.3%	35.4%	54.1%	50.1%
+FT-KAN	48.9%	84.5%	34.5%	53.2%	50.3%

Reference

(1) Bharati, P.; Pramanik, A. Deep Learning Techniques—R-CNN to Mask R-CNN: A Survey. In *Computational Intelligence in Pattern Recognition*; Das, A. K., Nayak, J., Naik, B., Pati, S. K., Pelusi, D., Eds.; Advances in Intelligent Systems and Computing; Springer Singapore: Singapore, 2020; Vol. 999, pp 657–668, DOI: 10.1007/978-981-13-9042-5_56

(2) Li, G.; Zeng, J.; Peng, Z.; Liang, Y.; Zheng, X.; Wang, T. E2EC: Edge-to-Edge Collaboration for Efficient Real-Time Video Surveillance Inference. *IEEE Transactions on Mobile Computing* **2025**, *24* (9), 9126–9140, DOI: 10.1109/TMC.2025.3559919

(3) Yu, D.; Yan, Z.; Ming, B. Real-Time Instance Segmentation Tracking Algorithm in Mixed Reality. In *2021 IEEE 7th International Conference on Virtual Reality (ICVR)*; IEEE: Foshan, China, 2021; pp 368–372, DOI: 10.1109/ICVR51878.2021.9483810

(4) Bi, J.; Zhu, Z.; Meng, Q. Transformer in Computer Vision. In *2021 IEEE International Conference on Computer Science, Electronic Information Engineering and Intelligent Control Technology (CEI)*; Fuzhou, China, 2021; pp 178–188, DOI: 10.1109/CEI52496.2021.9574462

(5) Hussain, M. YOLO-v1 to YOLO-v8, the Rise of YOLO and Its Complementary Nature toward Digital Manufacturing and Industrial Defect Detection. *Machines* **2023**, *11* (7), 677, DOI: 10.3390/machines11070677

(6) Wang, Z.; Zheng, L.; Liu, Y.; Li, Y.; Wang, S. Towards Real-Time Multi-Object Tracking. In *Computer Vision – ECCV 2020*; Vedaldi, A., Bischof, H., Brox, T., Frahm, J.-M., Eds.; Springer

International Publishing: Cham, 2020; pp 107–122, DOI: 10.1007/978-3-030-58621-8_7
(7) Zhang, Y.; Wang, C.; Wang, X.; Zeng, W.; Liu, W. FairMOT: On the Fairness of Detection and Re-identification in Multiple Object Tracking. *Int J Comput Vis* **2021**, *129* (11), 3069–3087, DOI: 10.1007/s11263-021-01513-4
(8) Wojke, N.; Bewley, A.; Paulus, D. Simple Online and Realtime Tracking with a Deep Association Metric. In *2017 IEEE International Conference on Image Processing (ICIP)*; Beijing, China, 2017; pp 3645–3649, DOI: 10.1109/ICIP.2017.8296962
(9) Zhang, Y.; Sun, P.; Jiang, Y.; Yu, D.; Weng, F.; Yuan, Z.; Luo, P.; Liu, W.; Wang, X. ByteTrack: Multi-Object Tracking by Associating Every Detection Box. In *Computer Vision – ECCV 2022*; Avidan, S., Brostow, G., Cissé, M., Farinella, G. M., Hassner, T., Eds.; Lecture Notes in Computer Science; Springer Nature Switzerland: Cham, 2022; Vol. 13682, pp 1–21, DOI: 10.1007/978-3-031-20047-2_1
(10) Jiang, B.; Zhang, Y.; Luo, B.; Cao, X.; Tang, J. STGL: Spatial-Temporal Graph Representation and Learning for Visual Tracking. *IEEE Trans. Multimedia* **2021**, *23*, 2162–2171, DOI: 10.1109/TMM.2020.3008035
(11) Stanojevic, V. D.; Todorovic, B. T. BoostTrack: Boosting the Similarity Measure and Detection Confidence for Improved Multiple Object Tracking. *Machine Vision and Applications* **2024**, *35* (3), 53, DOI: 10.1007/s00138-024-01531-5
(12) Somvanshi, S.; Javed, S. A.; Islam, M. M.; Pandit, D.; Das, S. A Survey on Kolmogorov-Arnold Network. *ACM Comput. Surv.* **2026**, *58* (2), 1–35. https://doi.org/10.1145/3743128.
(13) Chen, F.; Wu, F.; Xu, J.; Gao, G.; Ge, Q.; Jing, X.-Y. Adaptive Deformable Convolutional Network. *Neurocomputing* **2021**, *453*, 853–864, DOI: 10.1145/3743128
(14) Wu, J.; Shi, Y.; Wang, W. Fault Imaging of Seismic Data Based on a Modified U-Net with Dilated Convolution. *Applied Sciences* **2022**, *12* (5), 2451, DOI: 10.3390/app12052451
(15) Niu, Y.; Fan, S.; Cheng, X.; Yao, X.; Wang, Z.; Zhou, J. Road Crack Detection by Combining Dynamic Snake Convolution and Attention Mechanism. *Applied Sciences* **2024**, *14* (18), 8100, DOI: 10.3390/app14188100
